# Editorial: The State of the Art in Creative Arts Therapies

**DOI:** 10.3389/fpsyg.2020.00068

**Published:** 2020-02-05

**Authors:** Tal Shafir, Hod Orkibi, Felicity Anne Baker, David Gussak, Girija Kaimal

**Affiliations:** ^1^The Emili Sagol Creative Arts Therapies Research Center, University of Haifa, Haifa, Israel; ^2^The School of Creative Arts Therapies, University of Haifa, Haifa, Israel; ^3^Faculty of Fine Art and Music, University of Melbourne, Melbourne, VIC, Australia; ^4^The Graduate Art Therapy Program, Florida State University, Tallahassee, FL, United States; ^5^Department of Creative Arts Therapies, Drexel University College of Nursing and Health Professions, Philadelphia, PA, United States

**Keywords:** dance movement therapy (DMT), art therapy, music therapy (MT), drama therapy (DT), psychodrama, creative arts therapies research

Creative Arts Therapies is an umbrella term for healthcare professions that use the creative and expressive process of art making to improve and enhance the psychological and social well-being of individuals of all ages and health conditions. Creative arts therapies use the relationship between the client and therapist and among clients in group or dyadic therapy in the context of the creative-expressive process as a dynamic and vital force for growth and change. The creative-expressive process engages physiological sensations, emotions, and cognition; facilitates verbal and non-verbal symbolization, narration, and expression of conscious or unconscious conflicts and meaning-making through internal and external dialogue and communication between oneself and others.

The major objective of this Research Topic was to introduce, collect, discuss, and disseminate new clinical practices, scientific evidence, methodologies, theoretical concepts, and notions about Creative Arts Therapies. By publishing this open-access articles under this Research Topic we hope not only to distribute updated knowledge among the many clinicians in this field, but also to inform and convey the importance and significant therapeutic impact of this field, to scientists and clinicians from other psychological disciplines.

Creative arts therapists work in a variety of settings such as hospitals, educational institutions, community mental health facilities, prisons, hospices, and private practices, and include a variety of Professional specializations. Contributors to this Research Topic included experts in dance-movement therapy (DMT), drama-therapy and psychodrama, film therapy, music therapy, and art therapy. The topics of their studies vary from theoretical concepts and underlying mechanisms through methodology and up to evidence-based clinical studies and their review or meta-analysis. In the following paragraphs we summarized the 36 different contributions to this Research Topic, based on their artistic modality.

## Dance-Movement Therapy

Seven articles contributed to the modality of dance-movement therapy.

Payne and Brooks, wrote a theoretical article “Different Strokes for Different Folks: The BodyMind Approach as a Learning Tool for Patients with Medically Unexplained Symptoms to Self-Manage” in which, based on research and their DMT practice with patients with medically unexplained symptoms, they proposed a new approach to treat this population: The BodyMind Approach. A description of the theoretical underpinnings and philosophy of the proposed alternative to current interventions is presented as well as a description of the suggested intervention which incorporates creative arts therapies and adult learning techniques for self-management practices.

In their article “How Do We Recognize Emotion from Movement? Specific Motor Components Contribute to the Recognition of Each Emotion” Melzer et al., report a scientific study whose aim was to investigate the mechanism underlying DMT practices. In this study Melzer et al., demonstrated that the specific Laban motor components which were found in their earlier study (Shafir et al., 2016) to enhance specific emotions when moved, enable recognition of the same emotions when being observed, even when the mover didn't try to express any emotion. This study supports the notion of the existence of associations in the brain between certain movement components and specific emotions, a notion which can explain how internal simulation by the mirror neurons of observed movements can create empathy in the observer and help therapists to understand their clients' emotional state, by eliciting a similar emotion to that which is elicited in the client who moves with those movement component.

In another article: “How Shall I Count the Ways? A Method for Quantifying the Qualitative Aspects of Unscripted Movement With Laban Movement Analysis” the same group (Tsachor and Shafir) describe in details the methodology they had used in their 2016 study (Shafir et al., 2016) to narrow down and select out of many movement components the ones they used as variables for the statistical analysis with which they determined which movement components enhance which emotion.

Another article which deals with methodology, although not methodology of the study but that of intervention assessment, is the article by Dunphy K. F. et al., “Outcome-Focused Dance Movement Therapy Assessment Enhanced by iPad App MARA.” This article describes the applicability and benefits of using the iPad app MARA (Movement Assessment and Reporting App; Dunphy et al., 2016) to assess and report the progress of clients with intellectual disability as a result of a 16 weeks DMT program.

Three of the DMT articles reviewed the evidence for therapeutic effectiveness of this modality. While two articles reviewed the effectiveness of DMT on specific populations using mainly a qualitative synthesis: Karkou et al., examined the effectiveness of DMT in the treatment of adults with depression, and Goodill examined accumulating evidence for DMT effectiveness in Cancer care, the article which was published last in this Research Topic summarized nicely the overall effectiveness of this field. In their paper “Effects of Dance Movement Therapy and Dance on Health-Related Psychological Outcomes. A Meta-Analysis Update” Koch et al., conducted a very detailed meta-analysis (including a sensitivity analysis, assessment of heterogeneity, analysis of outliers and publication bias and analysis of follow-up data) on the effects of 21 DMT and 20 dance controlled intervention studies (2,374 participants) published between 01/2012 and 03/2018, on health-related psychological outcomes. They found in total a medium significant overall effect for dance and DMT intervention based on heterogeneous results. Since type of intervention was a significant source of heterogeneity, they explored the effects of DMT and dance separately and found that DMT consistently and with a high homogeneity significantly improved affect-related psychological conditions by decreasing anxiety and depression levels, and significantly increased quality of life and interpersonal and cognitive skills, whereas dance interventions increased (psycho-)motor Skills.

## Drama Therapy, Psychodrama and Film Therapy

One drama therapy, one film therapy, and eight psychodrama studies are featured in this special topic.

Drama therapist Feniger-Schaal et al. report on the application of the mirror game to assess the embodiment of attachment in adulthood. Associating attachment scores with non-verbal movement interactions constitutes the first step toward validating the mirror game as a standardized assessment tool in drama therapy and dance movement therapy.

Azoulay and Orkibi report the results of a mixed method study on first year MA students' psychodrama field training experience in Israel. The results point out possible helpful and hindering factors in students' field training and trajectories in their perceived professional identity and suitability, all of which may inform the design of field training in psychodrama programs.

Cruz et al. conducted a systematic review of psychodrama techniques implemented in research and practice. The results provide an inventory of operationalized definitions of core psychodrama techniques that was confirmed through consensus by international psychodrama experts and will be of value to researchers and trainers.

Ron's case study of an open psychodrama group in a psychiatric inpatient ward in Israel highlights how the doubling technique and the group sharing phase reinforce empathy, relatedness, and support, which may offer psychiatric inpatients relief from distress and loneliness.

A study by Gonzalez et al. elegantly illustrate how to implement the mixed methods hermeneutic single case efficacy design to explore treatment effectiveness. The quantitative results generally suggest positive changes in clients' self-identified problem, symptoms, and spontaneity, while the qualitative results underpin the attribution of these changes to the treatment.

Bucută et al.'s mixed methods study probes how psychodrama methods and techniques can empower abused women and promote changes from their victim role. The findings and discussion may inform readers interested in psychodramatic gender violence interventions.

The quantitative results of Testoni et al. suggest that a death education course with psychodrama and movie making activities helped high school students in Italy to work through a case of suicide. Enhanced sense of life meaning and reduced death anxiety were among the findings related to the processing of death related trauma and grief.

Filmmaking was also used by Tuval-Mashiach et al. in their qualitative study on Israeli military veterans suffering from service-related trauma. The results indicate that the “I Was There” video therapy program contributed to alleviating participants' trauma processes and sense of agency and affiliation.

In his theoretical article, Yaniv draws on the neurocognitive concept of bottom-up/top-down processing to explicate the somewhat enigmatic state of spontaneity or “trusting the process” in psychodrama. He reviews the scientific evidence in support of J. L. Moreno's contention that all individuals can learn to let go of predetermined top-down conceptions and be open to bottom-up processing of experiences in the here-and-now.

Sang et al. provide a historical analysis of the spread and development of psychodrama in mainland China. This article identifies key actors and processes that led to the development of three major branches of psychodrama in that country.

## Music Therapy

Two research studies were reported for music therapy. The first by Baker et al. was an interpretative phenomenological analysis of interviews with people with acquired neurodisabilities who had engaged in a songwriting program aimed at reconstructing a post-injured identity. Results of the analysis indicated that participants traveled through one of four recovery journeys. Some experienced their acquired injury as an opportunity for new beginnings, some were drawing on resilience from previous traumas to activate well-developed coping strategies, while others used the process of creating songs to identify new way of being in the world.

A study by Clark et al. of people living with dementia and their family caregivers focused on how therapeutic group singing enables these community dwelling older people to flourish. Interviews with participants revealed that the singing groups not only enhanced relationships between person with dementia and his or her family carer, but facilitated the development of new relationships with others attending the group. Participants also reported feeling more socially accepted and confident, experienced a lift in mood and an enhanced sense of purpose.

## Art Therapy

Thirteen articles, a third of all of the peer-reviewed articles represented in this Research Topic of *The State of the Art in Creative Arts Therapies*, focused on art therapy. Many of the articles, recognizing the need to invest in and develop robust yet quite varied research agendas, were dedicated to how the arts, art meaning, and aesthetic interactions can bring about positive and sustained change. Gerber et al. relied on a robust qualitative research agenda to explore aesthetic and intersubjective phenomena in the creative arts therapies and how such therapeutic approaches can transform perception, behavior, relationship and well-being. Their study “Arts-based research approaches to studying mechanisms of change in the Creative Arts Therapies” relied on a “…deductive thematic analysis of written accounts of simulated arts therapies experiences…” to determine the potential for complex transformative phenomena “that occur in the nexus of art-based expression, reflection and relationships.”

Focusing specifically on the need for empirical evidence on the therapeutic potential of art materials, Haiblum-Itskovitch et al.'s article, “Emotional response and changes in heart rate variability following art-making with three different art materials,” addressed how three different art materials that varied greatly in levels of fluidity–pencil gouache, and oil pastels–elicit various emotive responses and changes. This important scientific study relied on a combination of data from self-reports and an electrocardiogram device to inform their findings. Another empirical study, Zeevi et al.'s “The efficiency of art-based interventions in parental training” differed in tone and focus. The authors, in providing 87 parents two questionnaires before and after 10 months of art therapy treatment for their young children, while the children and 14 art therapists completed two questionnaires, assessed the difference between those parents who received parental training with art-based interventions, verbal training or no training at all. Also focusing on the relationship between parents and their children, Gavron and Mayseless's “Creating art together as a transformative process in parent-child relations: The therapeutic aspects of the joint painting procedure,” employed a qualitative method–as part of a much larger mixed-methods study–in which to ascertain the specific benefits of engaging in a specific art task to positively affect the relationship between 87 mother-child dyads.

Huss and Samson instituted a large-scale qualitative method to clarify the relationship between coping and art therapy, particularly the components of meaning, manageability, and comprehensibility, for those experiencing health-stress from cancer. In their study, “Drawing on the arts to enhance salutogenic coping with health-related stress and loss” they discovered that the arts naturally embodied the mechanisms that enhance and contain these components. As a natural extension of these positive results, they provide a protocol in how art can be used to enhance coping with such stressors. Nagamey et al. relied on an interpretive phenomenological analysis of semi-structured interviews and drawings to explore “Perspectives on social suffering…” specific to Palestinian adults who must cross a particular checkpoint into Israel for school and work. While regionally focused, their results could lead to a greater understanding of the social stressors by those experiencing political conflict around the world.

In addition, Binson and Lev-Weisel, relied on a phenomenological methodology to explore the benefits of applying experiential learning to facilitate personal and professional growth in doctoral students in Thailand attending academic lectures. As indicated in their article “Promoting personal growth through experiential learning…” they discovered that “…the experiential learning element within the course contributed to their personal well-being, improvements in their family and spousal relationships, enhanced social skills, as well as a changed self-perception in roles as lecturers and therapists.”

There has been ever increasing debate over the years of the benefits of examining the formal elements within the art over content as assessable indicators. Pénzes et al. relied on a constructivist grounded theory approach to examine how art therapists may use the formal elements of a drawing to better understand the mental health of their client. In their article “How art therapists observe mental health using formal elements in art products: Structure and variation as indicators for balance and adaptability” the authors interviewed eight art therapists and determined that rather than contribute to an understanding of a client's symptoms or diagnosis, such characteristics inform the balance and adaptability of the artist. However, in their article “Associations between perception of parental behavior and ‘Person Picking an Apple from a Tree’ drawings among children with and without special education needs,” Or et al. relied on the symbolic content of the Person Picking an Apple from a Tree drawing. Long associated with the Formal Elements Art Therapy Scale, rather than focus merely on *how* the drawings were completed, the authors relied on the content elements to quantitatively determine children's perceptions of parental behavior, which seemed much more revealing with those children with special needs.

It is imperative that research endeavors in art therapy be reexamined to ensure rigor, application of current theories as well as efficacy and viability of methodological approaches. In “Effectiveness of art therapy with adult clients of 201–What progress has been made,” Regev and Cohen-Yatziv examined the last 27 published studies in the field that examined the efficacy of art therapy with adult clients among an array of seven specific categories. In doing so, the authors have continued the necessary dialogues instrumental in furthering our own examinations within the field. Feen-Calligan et al., in “Art therapy, community building, activism, and outcomes” provided a descriptive study that examined the interrelationships that developed amongst graduate art therapy students who were tasked to prepare undergraduate service-learning students as part of their research class with the directors of six community agencies preparing for such students. Noting the growing trend of such hands-on practice in the community, the authors recognized the value of such an examination to inform other art therapy programs who hope to rely on service-learning to teach research.

As we continue to advance research in the field of art therapy, there has expanded a greater acceptance of the need to think outside the box, to go beyond the limitations of just our field, to rely on new innovations and push the envelope to provide the best services. In her article “Summary of twenty-first century great conversations in art, neuroscience and related therapeutics,” King emphasized the need for transdisciplinary collaboration to best understand the complexity of mental and physical disorders. Proposing that this article serves as a potential missing link to fill the gap amongst varied fields, King recounts a symposium at her home institution that brought together several divergent thinkers from a wide array of fields who were tasked to help develop a common language in which to advance the interplay of the creative arts therapies and neurosciences. And finally, in “The principles of art therapy in virtual reality,” Hacmun et al. take us well-beyond the boundaries of our physical reality and offer various perspectives on the potentials and challenges of using virtual reality in the therapeutic milieu, outlining much needed principles of its use.

## Multiple therapies

Two articles reviewed the effectiveness of interventions using different modalities of arts therapies. Lo et al. reported a qualitative systematic review of 11 (six music therapy, three visual art therapy, one DMT, and one applied literature therapy) creative arts-based intervention studies for stroke survivors. The authors identified five analytical themes: functional restoration, psychological support, social engagement, spiritual experience and short-comings and barriers, and concluded that overall art-based therapies have demonstrated strengths in addressing psychosocial needs for stroke survivals and that different art modalities are perceived to be useful in achieving different therapeutic goals.

Dunphy K. et al. examined the outcomes of four creative arts modalities (art, dance, drama, and music) interventions for older adults experiencing depression. In their review they also payed attention to the processes documented in those studies as contributing to the change, as well as the mechanisms considered to underlie these processes. Their analysis of 75 articles (17 art, 13 dance, 4 drama, and 41 music) indicated mostly significant qualitative or positive qualitative findings, where the mechanisms considered to contribute to the reduced depression included physical (e.g., increased muscle strength), intra-personal (e.g., enhanced self-concept; processing and communication of emotions), cultural (e.g., creative expression, aesthetic pleasure), cognitive (e.g., stimulation of memory), and social (e.g., increased social skills and connection) mechanisms.

Taken separately, each of the articles in this Research Topic provides a glimpse into the unique, complex, and far-reaching endeavors of members of our field. Together, the articles reflect not only the increasing evidence for the effectiveness of arts therapies interventions, but also the increasing diversity of perspectives as well as methodological sophistication in the field of arts therapies research, offering directions for how we might build on these foundations in the future.

## Author Contributions

All authors contributed to writing up the editorial.

### Conflict of Interest

The authors declare that the research was conducted in the absence of any commercial or financial relationships that could be construed as a potential conflict of interest.
